# Effect of diabetes mellitus on long-term outcomes after repeat drug-eluting stent implantation for in-stent restenosis

**DOI:** 10.1186/s12872-016-0445-6

**Published:** 2017-01-06

**Authors:** Lin Zhao, Weiwei Zhu, Xiaojiang Zhang, Dongfang He, Chengjun Guo

**Affiliations:** Department of Cardiology, Beijing Anzhen Hospital, Capital Medical University, 100029 Beijing, China

**Keywords:** Diabetes mellitus, Coronary restenosis, Percutaneous coronary intervention

## Abstract

**Background:**

Whether diabetes mellitus (DM) is a predictor of long-term adverse clinical outcomes after repeat drug eluting stent (DES) implantation for DES in-stent restenosis (ISR) remains controversial. We sought to evaluate the effect of DM on the long-term clinical outcomes in patients undergoing repeat DES implantation for DES-ISR lesions.

**Methods:**

In the present study, 254 patients with DES-ISR were divided into DM or non-DM groups according to the presence or absence of DM. All patients received repeat 2^nd^ generation DES implantation for DES-ISR. The occurrences of major adverse cardiac events (MACEs) over a 2-year follow-up period were compared between the two groups. MACEs were defined as cardiac death, myocardial infarction (MI), and target lesion revascularization (TLR). MACE free survival was investigated with Kaplan-Meier curve analysis. Cox regression analysis was used to identify factors associated with MACEs.

**Results:**

Baseline clinical characteristics were similar between groups, except for the prevalence of early restenosis (lower) in the DM group. Differences in angiographic and procedural characteristics were not significant between groups. The rates of 2-year MACE (30.9 vs. 26.0%; *P* = 0.453) and TLR (24.7 vs. 19.7%; *P* = 0.411) were similar between groups. MACE-free survival and TLR-free survival were also similar between groups (*P* = 0.441 and *P* = 0.807). Subgroup analysis suggested a significant difference in the MACE (39.0 vs.15.3%, *P* < 0.001) and TLR occurrence (30.5 vs.8.2%, *P* < 0.001) and TLR-free survival (lower in early subgroup, *P* < 0.001) between early and late occurrence of ISR in the non-DM group of patients but not in the DM group. After adjustment for all significant clinical variables, Cox regression analysis indicated that DM was not associated with MACEs (hazard ratio [HR] 1.531, 95% confidence interval [CI] 0.882-2.658, *P* =0.130). Non-focal type ISR and early ISR were predictors of MACEs (HR 2.671, 95% CI 1.468-4.858,*P* = 0.001; HR 4.703, 95% CI 2.725-8.117, *P* < 0.001, respectively).

**Conclusions:**

Patients with DM have similar 2-year clinical outcomes to patients without DM when repeat 2^nd^ generation DES was used for treatment of DES-ISR. DM is not the predictor of long-term prognosis in patients undergoing repeat 2^nd^ generation DES for DES-ISR.

## Background

Drug eluting stents (DES) widely used in clinical practice have dramatically reduced subsequent repeat target lesion revascularizations (TLR) after percutaneous coronary intervention (PCI) [1, 2]. Nevertheless, it is far away from completely eliminating in stent restenosis (ISR). Current therapeutic strategies to address ISR include plain balloon angioplasty, cutting balloon angioplasty, repeat DES implantation, radiation therapy, and drug coated balloon (DCB) angioplasty. Among these strategies, repeat DES implantation and DCB angioplasty are the most effective methods for treatment of ISR [3]. Repeat DES implantation is the most preferred modality because DCB is not available in our hospital.

Diabetes mellitus (DM) has been an established predictor of TLR and major adverse cardiac events (MACEs) after PCI for coronary de novo lesions [4, 5]. The biologic effects of DM include leading to plaque growth, vascular instability and risk of thrombosis [4]. However, in DES era some studies have suggested that DM has no longer correlated with ISR [6, 7], while others still identify DM as a predictor of TLR [8]. Overall it is likely that DM is not a predictor of ISR for vein grafts and simple lesions (ACC/AHA type A/B1 lesions) and unprotected left main coronary artery lesions [5, 9, 10].

In the 2^nd^ generation DES era, whether DM remains a predictor of long-term adverse clinical outcomes after repeat DES implantation for DES-ISR is controversial [6–8]. Resolving this question would help cardiologists choose the optimal therapy for these patients.

In order to evaluate the effect of DM on the long-term clinical outcomes in a consecutive series of patients undergoing repeat DES implantation for DES-ISR lesions, we compared 2-year MACEs between patients with DM and patients without DM from our PCI registry. The aim of this study was to discover whether DM is a predictor for MACEs in patients undergoing repeat DES after DES-ISR.

## Methods

### Patients

This study was a retrospective analysis of prospectively enrolled patients. The study population included 254 consecutively enrolled patients treated between January 2011 and March 2013 from the department of Cardiology, Beijing Anzhen Hospital, Capital Medical University’s PCI registry with previous PCI undergoing repeat DES implantation for ISR. Written informed consent was obtained from all patients. The institutional review boards at Beijing Anzhen Hospital and Research Institute of heart, lung and vascular disease (Beijing, China) approved this study.

Patients with the following criteria were included in this study. Patients with ISR defined as more than 50% diameter stenosis quantified by quantitative coronary angiography (QCA) in addition to myocardial ischemia and restenosis of a DES implanted for a de novo lesion. Myocardial ischemia was evaluated based on patients’ symptoms or noninvasive tests (stress electrocardiography and stress myocardial scintigraphy). Careful evaluation was performed by the clinical staff for the requirement to undergo repeat DES. Patients with the following conditions were excluded: Recurrent restenosis meaning lesions in graft vessels that were not ISR, thrombotic lesions suggested by angiographic findings.

### Study design

According to the presence or absence of DM all patients were divided into 2 groups (DM group and non-DM group). DM was defined as either a previous diagnosis of DM from the patient’s clinical history that had been treated with diet, oral agents, peptide analogs or insulin, or a new diagnosis during hospitalization according to the World Health Organization guidelines of fasting plasma glucose ≥ 7.0 mmol/l (126 mg/dl) or 2–h plasma glucose ≥ 11.1 mmol/l (200 mg/dl) [11].

Either everolimus-eluting stents (EES, Xience V; Abbott Vascular, Santa Clara, California, USA) or zotarolimus-eluting stents (ZES, Resolute; Medtronic Inc., Santa Rosa, California, USA) were implanted for treatment of ISR lesions.

A radial or a femoral approach was used for PCI using a 6 Fr or larger guide catheter to facilitate subsequent QCA. No bias was introduced in the choice of interventional approaches and DES. All patients provided informed consent for undergoing the procedure. For each case, the interventional physician remains the same with over ten years’ experience of PCI. Aspirin (300-mg loading-dose followed by 100 mg/day) was taken immediately after diagnosis with initial stenosis and thereafter. After PCI, patients received antiplatelet therapy with clopidogrel (75 mg/day following loading dose of 300 mg) for at least 1 year.

QCA was performed using an automatic edge-detection system (GE Co., California, USA). Coronary angiograms were analyzed at the angiographic core laboratory by trained personnel blinded to the group allocation. QCA measurements were obtained on both an in-stent basis (confined to the stented region) and an in-segment basis (including the vessel 5 mm proximal and distal to the stent). Lesion morphology was evaluated following The American College of Cardiology/American Heart Association (ACC/AHA) classification. Lesion length was measured from QCA measurements before PCI. Calcification was assessed by coronary angiography and qualitatively classified as none, mild, moderate, or severe. Angulation was quantified by measuring the maximal angle in the target lesion before PCI. The vessel angulation was classified as minor (<45°), moderate (45–90°), and severe (>90°). Ostial lesions were within 3 mm of the coronary ostium or 3 mm distal to a major proximal side branch. A diameter of stenosis greater than 50% involving the main parent vessel, with or without involvement of side branch ostium, or the origin of the side branch was defined as bifurcation lesion. We classified the types of restenosis into focal type (<10 mm in length) and non-focal type, which included diffuse (restenosis > 10 mm within the stent), proliferative (restenosis >10 mm in length extending outside the stent), and occlusive. At the same time, they were also classified into early type (found any time in 1 year after DES implantation) and late type (found any time after 1 year after DES implantation).

## Clinical data collection

A dedicated data coordinating center performed all data management and analyses. Pre-specified clinical and demographic data, as well as clinical events during hospitalization, were collected from the hospital charts, reviewed by qualified personnel blinded to the objectives of the study, and entered prospectively into the database. Long-term clinical outcomes were conducted by clinic visit.

Data was collected for the occurrence of MACEs, defined as the combined incidence of death, MI, and TLR [12]. Death was defined as mortality from cardiac cause. MI was defined as typical ischemic chest pain and/or ST-segment and/or T-wave abnormalities with a creatine kinase isoenzyme-MB increase 2 times the reference values. We defined TLR as clinically driven revascularization of index lesion. A residual stenosis of <30% with TIMI-3 flow was defined as PCI angiographic success. Angiographic success without TLR, Q wave MI, or death prior to hospital discharge was defined as clinical success. We defined Chronic renal insufficiency (CRI) as patients receiving medical therapy or dialysis for chronic renal failure or have a creatinine level 2.0 mg/dL on admission.

## Statistical analysis

All statistical analyses were performed using the SPSS statistical software package (version 17.0; SPSS Inc., Chicago, Illinois, USA). A normality test demonstrated a normal distribution of the data. We presented continuous variables as mean ± SD while categorical variables as frequencies (%). Unpaired Student’s *t*-test was used for comparison of continuous variables and categorical variables was compared using *χ* ^2^ or Fisher’s exact tests. A Kaplan–Meier curve was plotted for the MACE-free survival of both groups using GraphPad Prism (version 5; GraphPad Software Inc., La Jolla, California, USA). Difference between groups was evaluated by a log-rank test. A censored data indicated the loss of follow up. A Cox regression analysis was performed to identify whether there is independent association between DM and higher incidence of MACEs. Variables were selected on the basis of overall clinical relevance, according to the previously published literature with particular attention paid to clinical and procedural factors that would make MACE more likely and included age, sex, hypertension, diabetes mellitus, chronic renal insufficiency, calcification lesion, bifurcation lesions, no-focal lesion and early restenosis. Statistical significance was defined as a *P* value less than 0.05.

## Results

### Baseline clinical characteristics

Among the 254 consecutive patients, 81 patients were diagnosed with DM. The baseline clinical characteristics of the patients are listed in Table 1. There were no significant differences in many coronary artery disease risk factors including hyperlipidemia, and smoking between the two groups. In the DM group, the mean hemoglobin A1c (HbA1c) and mean fasting plasma glucose level were higher than those in the non-DM group (HbA1c, 7.373 ± 1.216 vs. 5.463 ± 0.754%, *P* < 0.001, fasting glucose, 7.647 ± 2.416 vs. 5.586 ± 1.189, *P* < 0.001). Fewer cases of early restenosis occurred in the DM group than the non-DM group (24.8 vs.48.2%, *P* = 0.04).

**Table 1:** Baseline clinical characteristics

Variables	DM *N* = 81	Non-DM *N* = 173	*P*-value
Basic variables			
Age (years)	60.22 ± 10.36	59.42 ± 10.68	0.572
Male (%)	65 (80.2%)	136 (78.6%)	0.869
Smoking (%)	20 (24.7%)	44 (25.4%)	0.950
Prior diseases			
Hyperlipidemia (%)	38 (46.9%)	87 (50.3%)	0.687
Previous MI (%)	7 (8.6%)	22 (12.7%)	0.402
Hypertension (%)	37 (45.7%)	77 (44.5%)	0.632
Chronic renal insufficiency (%)	12 (14.8%)	25 (14.5%)	0.873
Clinical presentation			
Stable angina (%)	52 (64.2%)	99 (57.2%)	0.646
Unstable angina (%)	21 (25.9%)	48 (27.7%)	
NSTEMI (%)	5 (6.2%)	15 (8.7%)	
STEMI (%)	3 (3.7%)	11 (6.4%)	
Medication			
ACEI/ARB (%)	53 (65.4%)	119 (68.8%)	0.666
Dural antiplatelet therapy (%)	73 (90.1%)	149 (86.1%)	0.423
Statin therapy (%)	75 (92.6%)	151 (87.3%)	0.283
Lab parameters			
Fasting glucose	7.647 ± 2.416	5.586 ± 1.189	<0.001
Hemoglobin A1c	7.373 ± 1.216	5.463 ± 0.754	<0.001
Type of restenosis			
Early restenosis	27 (24.8%)	82 (48.2%)	0.04
Non-focal restenosis (%)	23 (28.4%)	64 (37%)	0.203

Data are presented as mean ± SD or n (%)

*MI* myocardial infarction, *NSTEMI* non-ST segment elevated myocardial infarction, *STEMI* ST segment elevated myocardial infarction, *ACEI* angiotensin-converting enzyme inhibitor, *ARB* angiotensin receptor blocker

### Angiographic and procedure characteristics

All procedures were successful, both angiographically and clinically, without any major complications. All patients were discharged home after the intervention at a mean duration of 2.3 ± 1.6 days. The angiographic and procedural characteristics are displayed in Table 2. The lesion-based angiographic and procedural characteristics were similar between the both groups with no significant differences.

**Table 2:** Angiographic and procedural characteristics

Variables	DM *n* = 81	Non-DM *n* = 173	*P*-value
Vessel			
LM/LAD/LCX/RCA	2/38/14/27	4/97/29/43	0.498
Type B2/C lesions	68 (84%)	133 (76.9%)	0.196
Lesions			
Lesion lengths, mm	25.81 ± 10.82	23.86 ± 11.02	0.187
Ostial location	9 (11.1%)	18 (10.4%)	0.865
Angulation > 45°	34 (42%)	58 (33.5%)	0.209
Moderate-severe calcification	16 (19.8%)	30 (17.3%)	0.727
Bifurcation	19 (23.5%)	49 (28.5%)	0.449
Repeat DES			
Type (EES/ZES)	37/44	69/104	0.414
Number of stents (n)	1.61 ± 0.92	1.75 ± 0.89	0.263
Stent length (mm)	27.21 ± 10.92	24.65 ± 10.83	0.082
Stent diameter (mm)	2.86 ± 0.41	2.93 ± 0.39	0.234

Data are presented as mean ± SD or n (%)

*LM* left main coronary artery, *LAD* left anterior descending coronary artery, *LCX* left circumflex artery coronary artery, *RCA* right coronary artery, *EES* Everolimus Eluting Stent, *ZES* Zotarolimus Eluting Stent

### 2-year clinical outcome

**Fig. 1:**
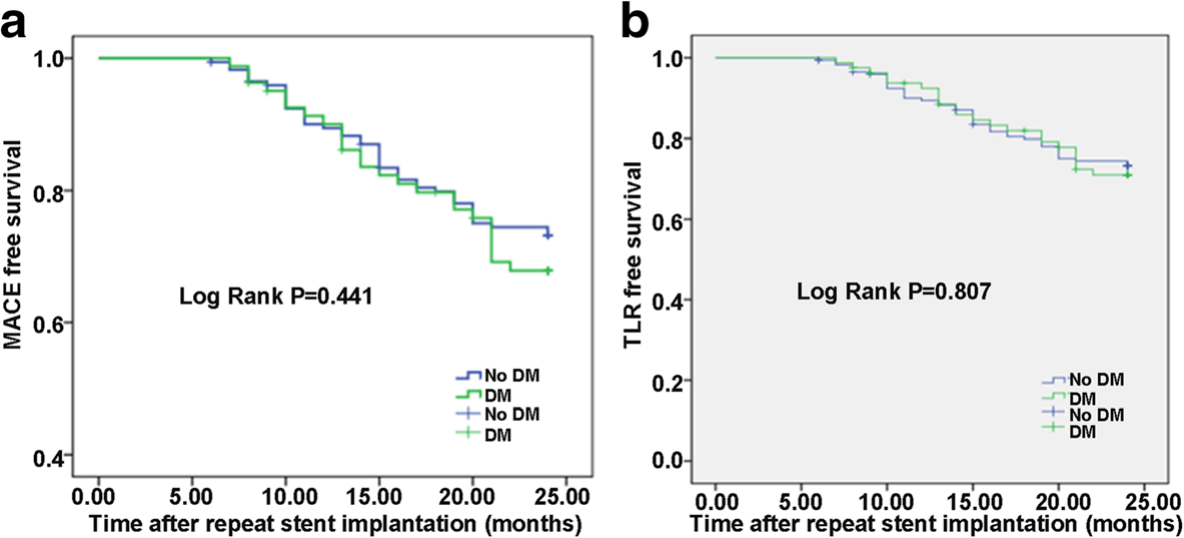
Kaplan–Meier curves of 2-year MACE-free and TLR-free survival

During the 2-year follow-up period, total 4 patients in DM group and 9 patients in non-DM group were lost to follow-up. As shown in Table 3, 25 MACEs were observed in the DM group and 45 in the non-DM group (30.9 vs. 26.0%; *P* = 0.453). TLR was required in 20 patients in the DM group and 34 in the non-DM group (24.7 vs. 19.7%; *P* = 0.411). Two patients in the DM group and 3 patients in the non-DM group died from all-cause mortality (2.5 vs.1.7%, *P* = 0.655). Three patients in the DM group and 8 patients in the non-DM group had MI (3.7 vs.4.6%, *P* = 0.996). The Kaplan–Meier curves showed that the DM group had similar MACE-free survival to the non-DM group with the log-rank test (*P* =0.441, Fig. 1a) and similar TLR-free survival (*P* = 0.807, Fig. 1b).

**Table 3:** Two-year outcomes

Events	DM group *n* = 81	Non–DM group *n* = 173	*P*-value
MACE	25 (30.9%)	45 (26%)	0.453
TLR	20 (24.7%)	34 (19.7%)	0.411
MI	3 (3.7%)	8 (4.6%)	0.996
Death	2 (2.5%)	3 (1.7%)	0.655

### Analysis of MACEs according to early- or late-ISR

**Fig. 2:**
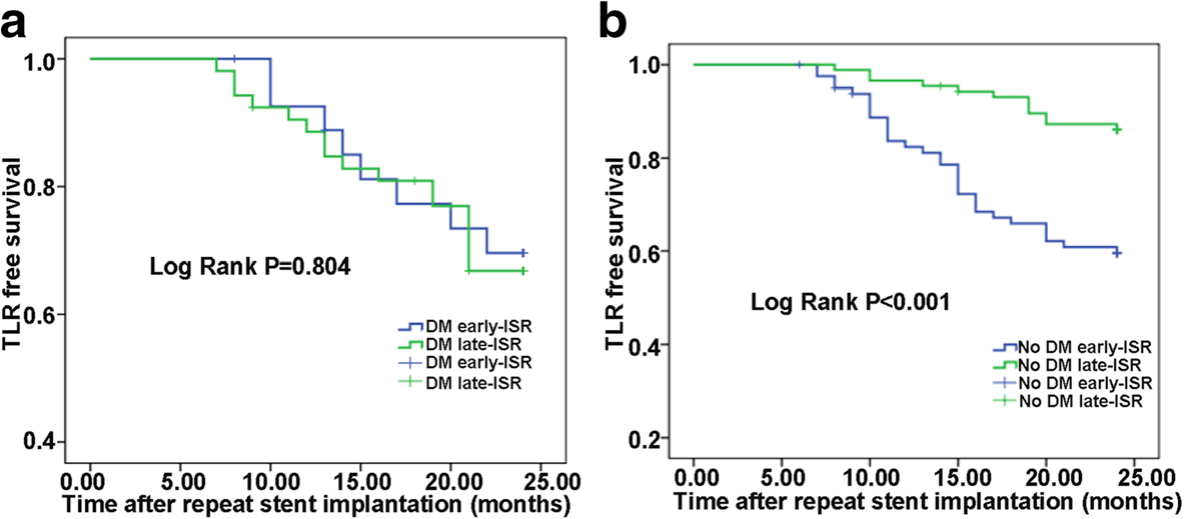
Kaplan–Meier curves of 2-year TLR-free survival in the early and late ISR subgroups. **a**: DM-ISR KM curve; **b**: Non DM-ISR KM curve

When stratification analysis was performed according to the occurrence of early-ISR or late-ISR in the two groups it became apparent that there was no significant difference in the occurrence of MACEs between early and late ISR subgroups in the DM group but in the non-DM group there was a significantly higher rate of overall MACEs and TLR in the early ISR subgroup than in the late ISR subgroup (both *P* < 0.001, Table 4), but rates of MI and death were similar. Kaplan-Meier curves supported this within the DM subgroups with similar TLR-free survival (*P* =0.804, Fig. 2a) and a significant difference was found between the non-DM subgroups for TLR-free survival (*P* < 0.001, Fig. 2b).

**Table 4:** The stratification of early-ISR and late-ISR groups

	DM-early-IS	DM-late-ISR		Non-DM-early-ISR	Non-DM-late-ISR	
	R *n* = 27	*n* = 54	*P*-value	*n* = 82	*n* = 85	*P*-value
Events						
MACE	8 (30.9%)	17 (26%)	0.712	32 (39.0%)	13 (15.3%)	<0.001
TLR	7 (25.9%)	15 (27.8%)	0.997	25 (30.5%)	7 (8.2%)	<0.001
MI	1 (3.7%)	1 (1.9%)	0.998	5 (6.1%)	4 (4.7%)	0.743
Death	0 (0.0%)	1 (1.9%)	0.817	2 (2.4%)	2 (2.4%)	0.971

### Factors that predict MACEs

Parameters used in the multivariable model to assess the predictors of 2-year MACEs included age, sex, hypertension, DM, chronic renal insufficiency, calcification lesion, bifurcation lesions, no-focal lesion and early restenosis. Cox regression result showed that non-focal lesion (hazard ratio (HR) 2.671, 95% confidence interval (CI) 1.468–4.858, *P* =0.001) and early restenosis (HR 4.703, 95%CI 2.725-8.117, *P* < 0.001) were independent predictors of MACEs among the nine variables included in the current study (Table 5).

**Table 5:** Predictors of 2-year MACEs by Cox regression analysis

Variables	Hazard ratio	95% confidence interval	*P* value
Sex	1.505	0.837	0.181
Age	0.989	0.964	0.381
Hypertension	0.915	0.546	0.737
Diabetes mellitus	1.531	0.882	0.13
Calcification	0.896	0.280	0.852
Bifurcation lesions	0.814	0.494	0.415
Non-focal lesions	2.671	1.468	0.001
Early restenosis	4.703	2.725	<0.001
Chronic renal insufficiency	1.748	0.579	0.322

## Discussion

The results reveal that there were no differences in the rate of 2-year MACEs in DM and non-DM groups in our study population (30.9% in DM group vs. 26% in non-DM group, *P* = 0.453).It indicates that DM is not a risk factor of poor long-term prognosis in patients undergoing repeat 2^nd^ generation DES implantation for DES-ISR (HR 1.531, 95%CI 0.882-2.658, *P* = 0.130).

Clinical use of DES has reduced the incidence of ISR, but treatment of DES-ISR is still challenging. [3, 13] The etiology of ISR remains unclear. Smooth muscle cell proliferation and neoatherosclerosis are the probable causes [14]. Clinical studies have proved that repeat DES implantation could acquire acceptable clinical and angiographic results for DES-ISR treatment [8–18]. Intravascular ultrasound (IVUS) or optical coherence tomography (OCT) can give us more important details of lesion characteristics of DES-ISR, which may improve the clinical results of repeat DES for DES-ISR treatment [19, 20]. Unfortunately, IVUS or OCT was not used for every patient enrolled in our study. During the 2-year follow-up of our study, 25 MACEs including 20 TLR were observed in the DM group and 45 MACEs including 34 TLR in the non-DM group. More TLRs occurred in the current study than previous studies [15–18]. In current study, follow-up period was 2 years which is relatively longer than that in previous studies [17, 18], which indicated that a longer follow-up period may contribute to the higher occurrence of TLR than before [14]. In addition, multivariable model analysis showed that non-focal and early ISR were the only two independent predictors of MACEs over the 2-year follow-up. Therefore, the angiographic type of DES-ISR, especially the non-focal type, and early phase of DES-ISR were highly associated with the TLR after repeat DES implantation. When our data was analyzed in terms of early and late occurrence of ISR we found that the differences in the rate of MACEs and TLR and TLR-free survival were significant only in the non-DM group. It is not clear why the occurrence of early ISR was not important for MACEs and in particular TLR in the DM group, but the DM subgroups were smaller than the non-DM subgroups so this may have influenced the analysis. Further studies are needed to investigate the mechanisms and better treatment methods for non-focal and early phase DES-ISR and whether these are influenced by DM.

It is well known that DM is a risk factor for MACE after PCI, but whether DM is still an independent predictor of ISR after PCI for all lesions is unknown in the DES era, especially in the 2^nd^ generation DES era. Previous studies have reported conflicting results [6–8]. Some of them comparing outcomes after PCI with 2^nd^ DES suggest that DM no longer correlates with ISR [6, 7], whereas others confirm that DM is still a risk factor of TLR [8]. Therefore, some investigators have concluded that DM is no longer a predictor of ISR after DES implantation for specific populations, such as patients with vein graft lesions, simple lesions (ACC/AHA type A/B1 lesions) and unprotected left main [5, 9, 10]. This study suggests that in 2^nd^ generation DES era, DM is not an independent risk factor of MACE in patients accepting repeat DES implantation for DES-ISR. The 2^nd^ generation DES is better in reducing ISR than the 1^st^ generation DES and BMS, which correlates with similar 2-year rates of TLR and overall MACE between DM and non-DM patients with ISR lesions in this study. On the other hand, ISR lesions are different from coronary de novo lesions [21], which maybe in part contribute to these results in these special patients. At the same time, our study reveals that non-focal type and early ISR independently predict the adverse outcomes after PCI for DES-ISR with 2^nd^ generation DES, which indicates that mechanical factors underlying non-focal type or early ISR other than DM maybe play important roles in the repeat occurrence of ISR after PCI for DES-ISR lesions [22–24].

### Limitation

Our study is a real-world, single-center, retrospective study about treatment of DES-ISR using 2^nd^ generation DES in DM patients. This study has some limitations. First, it is a retrospective study and biases inherent in this study may have substantially influenced the outcome of the results. However, in general, the groups were well balanced in baseline clinical, angiographic, and procedural characteristics. Other limitations of the present study include a small sample size in particular in the DM group, low use of OCT or IVUS in the total population, lack of routine angiographic follow-up, and no information on the selection of DES for the previous PCI because data was not available for patients treated at other hospitals for their first intervention. However, we did not find any significant differences between groups with regard to TLR, death and MI. In view of these limitations, our findings should be considered as a primary hypothesis generating data for larger studies that are needed to confirm the present findings.

## Conclusions

Long term outcomes equate between DM and non-DM groups of patients when a 2^nd^ generation DES was used for treatment of DES-ISR. DM is not a predictor of long-term prognosis in patients undergoing repeat 2^nd^ generation DES for DES-ISR.

## Abbreviations

ACC/AHA: The American college of cardiology/American heart association
BMS: Bare-metal stents
CRI: Chronic renal insufficiency
DCB: Drug coated balloon
DES: Drug eluting stent
DM: Diabetes mellitus
HbA1c: Hemoglobin A1c
HR: Hazard ratio
ISR: In-stent restenosis
IVUS: Intravascular ultrasound
MACEs: Major adverse cardiac events
MI: Myocardial infarction
OCT: Optical coherence tomography
QCA: Quantitative coronary angiography
TLR: Target lesion revascularization
